# Factors influencing self-report adherence to treatment in a sample of patients with hypertension in the west Pomeranian Voivodeship of Poland

**DOI:** 10.3389/fpubh.2025.1536430

**Published:** 2025-03-24

**Authors:** Izabela Cerzniewska, Edyta Gierycz, Kamila Rachubińska, Daria Schneider-Matyka, Ireneusz Walaszek, Dorota Ćwiek, Przemysław Ustianowski, Elżbieta Grochans, Anna Maria Cybulska

**Affiliations:** ^1^Department of Nursing, Pomeranian Medical University in Szczecin, Szczecin, Poland; ^2^Independent Public Health Care Center in Choszczno, Choszczno, Poland; ^3^Department of Obstetrics and Pathology of Pregnancy, Pomeranian Medical University in Szczecin, Szczecin, Poland

**Keywords:** adherence, compliance, concordance, management of adherence, hypertension, treatment recommendations

## Abstract

**Background/objectives:**

One of the major challenges in managing hypertension is non-adherence to treatment recommendations. This issue poses a significant barrier to effectively controlling blood pressure and preventing related cardiovascular complications. The main objective of this study was to demonstrate the level of adherence to therapeutic recommendations by hypertensive patients, and to determine how socio-demographic and medical variables affect adherence.

**Methods:**

The study was conducted among 205 patients with diagnosed hypertension hospitalized in the West Pomeranian Voivodeship. The study used a diagnostic survey method, a survey technique, and a self-administered questionnaire and the Adherence to Refills and Medication Scale.

**Results:**

The overall score for the ARMS questionnaire was 24.32, which is 2.03 points per question and indicates that adherence to therapeutic recommendations among the hypertensive patients surveyed was at a good level. Based on the collected data, it was shown that the older the age, the worse the adherence was. In addition, urban residents adhered to therapeutic recommendations less frequently than other respondents.

**Conclusion:**

Patients with diabetes were more likely to be non-compliant than patients without diabetes. And respondents with diagnosed CHF were more likely to adhere to recommendations than other respondents. Age, occupational activity and place of residence influenced adherence to treatment recommendations among hypertensive patients surveyed.

## 1 Introduction

Based on a World Health Organisation (WHO) report, an estimated 1.28 billion adults aged 30–79 years suffer from hypertension worldwide, with the majority of these people living in low- or middle-income countries (1–3). In addition, data published in 2017 by the NCD Risk Factor Collaboration show that the number of adults with elevated blood pressure increased from 594 million in 1975 to 1.13 billion in 2015, mainly in low- and middle-income countries, and in 2015, approximately 20% of women and 24% of adult men worldwide had hypertension (4). The highest proportion of men with hypertension was reported in central and eastern European countries - Croatia, Latvia, Lithuania, Hungary and Slovenia. On the other hand, the five countries with the highest percentage of women with hypertension are African countries: Niger, Chad, Mali, Burkina Faso and Somalia. European countries with the lowest percentage of adults with hypertension among men and women included the UK, Iceland, Cyprus, Ireland, Belgium and Switzerland. The highest rates of hypertension were observed in Croatia, Slovenia, Hungary, Latvia and Bulgaria, and for women in Bosnia and Herzegovina. Among the 41 countries analyzed, Poland ranked 32nd in terms of the percentage of adult men with hypertension, with a value of 38.1%, and 30th in terms of the percentage of adult women with hypertension, with a value of 30.6% (5).

In contrast, the prevalence of arterial hypertension in Poland, assessed on the basis of current criteria from the guidelines of the Polish Society of Hypertension, is 32.5% in adults under 80 years of age, while in the general population, including the older adult, it reaches approximately 35% (6). Epidemiological studies on hypertension have been conducted in Poland (5, 7, 8). The first of these were the Pol-MONICA surveys conducted in 1983/84, 1987/88 and 1992/93. In the following years, the NATPOL I (1994), NATPOL II (1997), NATPOL III PLUS (2002) and WOBASZ (2003–2005) surveys were conducted, which formed the basis for the POL-KARD study on cardiovascular disease risk. In 2011, the NATPOL 2011 study was conducted. Another study indicating the prevalence of hypertension is the PolSenior study (9). In addition, the WOBASZ II study was conducted in 2013–2014. One of the most recent studies on hypertension in Poland is the work of Malyszko et al. (10) conducted in 2017 as part of an international screening campaign on a sample of nearly 6,000 people.

Both the World Health Organization and the European Society of Cardiology (ESC) and the European Society of Hypertension (ESH) define hypertension as a cardiovascular disease that is associated with an overall elevated systolic blood pressure of ≥140 mmHg and/or a diastolic blood pressure of ≥90 mmHg (11, 12). Therefore, a key goal of hypertension therapy is to optimize blood pressure values and reduce cardiovascular events (13). There are non-pharmacological recommendations that include lifestyle changes, a balanced diet, moderate physical activity, reduction of alcohol consumption and smoking cessation. Pharmacological recommendations, on the other hand, include systematic intake of hypotensive medication as prescribed by the physician (14).

One of the major problems in contemporary cardiovascular care is non-adherence to medication, which is a significant barrier to preventing complications, optimizing blood pressure or reducing the cost of care for patients diagnosed with hypertension. Non-adherence to medical recommendations is a complex and multidimensional variable, consisting of three measurable phases: initiation, implementation and cessation of treatment (15).

Many studies suggest that high adherence to antihypertensive treatment recommendations is associated with better blood pressure control and reduced risk of cardiovascular disease (16–18). Understanding the factors influencing adherence to medical recommendations can play an important role in developing interventions aimed at improving it. Qualitative and quantitative studies described in the literature show that many factors potentially influence adherence to medical recommendations, including demographic, social, and cognitive factors, interactions between healthcare providers and patients, characteristics of the healthcare system, used medications, and the overall health profile of the patient (19–37). The World Health Organization has divided these factors into five categories: patient-related factors, socio-economic influences, health system factors, therapy-related factors and health status-related factors (20). Also, misinformation about the correct intake of medication or recommended diet, as well as disease monitoring, can lead to non-adherence to medical recommendations. Therefore, active involvement of patients diagnosed with hypertension can effectively improve their adherence to medical recommendations (35). A review of the literature indicates that, in order to achieve the planned health goals in hypertension therapy, the patient should adhere to the doctor’s prescription at least 80% of the time (36). On the other hand, irregular adherence to prescribed medication prevents good disease control, which negatively affects therapeutic outcomes. Compliance and adherence is the key to effective hypertension therapy (37, 38).

The literature review revealed that few researchers have analyzed the impact of various factors on adherence among patients diagnosed with hypertension living in Poland. Due to the difficulties faced by Polish retirees (socioeconomic disparities, long waiting times for specialists, high treatment costs, educational barriers, lack of education about hypertension or lack of effective screening), and considering the inadequacy of population-based hypertension treatment, the objective of this study was to assess the level of self-reported adherence to treatment in a group of patients with hypertension in the West Pomeranian Voivodeship of Poland. Moreover this study was to get a better understanding the sociodemographic and medical variables influencing adherence among these patients.

## 2 Materials and methods

### 2.1 Settings and design

For the present study, 245 hypertensive patients (primary hypertension ICD: I10) from the West Pomeranian Voivodeship who were hospitalized for hypertension were recruited.

A purposive selection method was used to select the study group, and the inclusion criteria were:

- Diagnosed hypertension (primary hypertension ICD: I10),
- Patients hospitalized in the Independent Public Health Care Facility in Choszczno at the Internal Medicine and Cardiology Department (for example: hypertensive crisis or heart failure) or follow-up visits of hypertensive patients from the Cardiology Clinic in Choszczno
- Duration of disease greater than 6 months,
- Age over 18 years,
- Hypertension treated with at least 1 antihypertensive drug,
- Voluntary consent of the respondent to participate in the study.

The exclusion criteria were: lack of consent to participate in the study; lack of understanding of the content of the questionnaires; and the patient’s clinical condition preventing them from completing the questionnaire on their own. Ultimately, 205 respondents who met all the inclusion criteria were involved in study (completion rate: 83,7%).

According to data from the National Health Fund, almost 10 million adult Poles will suffer from hypertension in 2020. The size of the study sample was determined on the basis of statistics on the hypertensive population in Poland in 2022 (5). The confidence level was set at 95%, the maximum error at 7% and the estimated fraction size at 0.5. The required number of people in the study was 196. The study was conducted in accordance with the Declaration of Helsinki after obtaining the opinion of Bioethics Committee of the Pomeranian Medical University in Szczecin.

Prior to the study, respondents were informed about the purpose of the study, the rules for completing the questionnaire and the maintenance of full confidentiality and anonymity among respondents. Respondents were also informed of the possibility to withdraw from participation at any stage of the research without giving any reason. The research was conducted using a printed survey questionnaire with a cover letter informing about the purpose of the research, how to complete it, the time needed, asking for honest and thoughtful answers. Survey questionnaires were filled out by patients. After a specified time, the completed questionnaires were collected by the interviewer.

### 2.2 Research methods and tools used

The research was carried out using the traditional method, by distributing a paper version of the questionnaire containing the author’s survey questionnaire and a standardized research tool - the ARMS (Adherence to Refills and Medication Scale) questionnaire - to nurses.

The Adherence to Refills and Medication Scale (ARMS) is a self-report questionnaire developed by Kripalani et al. (39) and later adapted and translated into Polish by Lomper et al. (40) to assess medication adherence in patients with chronic conditions, including hypertension. The questionnaire consists of 12 items that evaluate key aspects of medication therapy, such as adherence to the prescribed dose, medication timing, refilling prescriptions, and maintaining a continuous supply of medications. Responses are scored on a Likert scale ranging from 1 point (never) to 4 points (most of the time), with total scores ranging from 12 to 48 points, where higher scores indicate poorer adherence. Although the ARMS questionnaire does not provide specific cutoffs to categorize adherence as low or high, the average score per question can be calculated and interpreted based on the individual question’s scale. The scale is divided into two components: medication adherence (8 questions, scores ranging from 8 to 32 points) and medication and prescription refills (4 questions, scores ranging from 4 to 16 points). The ARMS-12 has demonstrated excellent internal consistency with a Cronbach’s *α* of 0.954, reflecting strong reliability (21).

The ARMS has been validated in multiple studies and proven effective in assessing medication adherence, showing robust construct validity. Overall, the ARMS is widely regarded as a reliable and valid tool for measuring medication adherence, with widespread use in both clinical practice and research.

The self-administered questionnaire consisted of questions covering sociodemographic data (age, gender, education, marital status, place of residence, occupational activity), as well as medical variables: those related to hypertension, those related to comorbidities (especially diabetes, renal failure, circulatory failure and stroke), stimulants (tobacco, alcohol), physical activity, adherence to dietary recommendations, factors aggravating the condition, the most frequent source of information about the disease and subjective assessment of quality of life.

### 2.3 Statistical analysis

Quantitative variables were characterized using descriptive statistics methods. For variables expressed on a quantitative scale, measures of central tendency (M - mean, Mdn - median), measures of variability (SD - standard deviation, IQR/2 - quarter deviation, CV - coefficient of variation) were determined. For variables expressed on a categorical scale, measures of structure were determined: abundance (N) and purity (%). The results of the standardized psychometric measures were recalculated according to the principles described by their creators and converted to a sten scale using norms for the Polish general population. Analysis of qualitative variables was performed by calculating the number and percentage fractions of occurrences of each value. In order to compare the values of quantitative variables in two groups, a nonparametric Mann–Whitney U test was performed. On the other hand, comparison of the values of quantitative variables in three or more groups was performed using the Kruskal-Wallis rank-sum non-parametric test. Once statistically significant differences were detected, post-hoc analysis was performed with the Dunn’s test to identify statistically significantly different groups. Correlations between quantitative variables were analyzed using the Spearman correlation coefficient. A default statistical significance level of 0.05 was assumed for all analyses. Calculations were performed using R v 4.2.2. statistical software (R Studio, Boston, MA, USA).

## 3 Results

### 3.1 Characteristics of sociodemographic and medical variables

The study included 205 patients diagnosed with hypertension. The mean age of the respondents was 60.61 years (SD = 10.9 years). The largest group consisted of women (60.98%), persons in a formal relationship (46.83%), living in cities of 10–100,000 inhabitants (40.00%), pensioners (70.73%) and persons diagnosed with hypertension 31–40 years ago (32.68%) (Table 1).

**Table 1:** Characteristics of the study group.

Variables (*N* = 205)		*n*	%
Gender	Men	80	39.02
	Women	125	60.98
Age [years]	Less than 60 years	42	20.49
	60–80 years	123	60.00
	Over 80	40	19.51
Marital status	Formal relationship	96	46.83
	Informal relationship	32	15.61
	Single	77	37.56
Place of residence	Village	41	20.00
	City with up to 10,000 residents	39	19.02
	City with 10,000–100,000 residents	82	40.00
	City more than 100,000 population	43	20.98
Professional activity	Economically active	47	22.93
	Inactive professionally	13	6.34
	Pensioner	145	70.73

The study observed that the most common comorbidities were diabetes (57.07%) and circulatory failure (20.98%). In addition, the vast majority of respondents were non-smokers (72.68%). Among smokers, the most common duration of cigarette smoking was 11–20 years (26.79%) or 21–30 years (26.79%). The majority of respondents did not do any physical activity (65.37%) and knew the dietary recommendations used by hypertensive patients (86.34%). It is worth noting that 14.15% of respondents strictly followed the recommended diet, 67.41% of people did not add salt to their food and 60.00% of people limited alcohol.

### 3.2 Adherence to therapeutic recommendations according to ARMS by patients with hypertension

The study analyzed adherence to treatment recommendations using the ARMS questionnaire. Based on the results collected, it was shown that the mean overall score for this questionnaire was 24.32 points, which is 2.03 points per question. This means that the average frequency of non-adherence is ‘rarely’. For the subscales ‘taking medicines’ and ‘refilling medicines and prescriptions’, the average score of respondents was 15.63 points and 8.69 points, respectively, meaning that the frequency of non-adherence to these recommendations is ‘rarely’.

### 3.3 Adherence to therapeutic recommendations according to ARMS by patients diagnosed with hypertension according to sociodemographic variables

This study evaluated the impact of sociodemographic variables (gender, age, marital status, place of residence, occupational activity) on adherence to treatment recommendations based on the ARMS questionnaire among patients diagnosed with hypertension. The study showed a statistically significant association between age and adherence to treatment among hypertensive patients. It was observed that age positively correlates with the ARMS total score, medication adherence and refill of medications and prescriptions. This means that the older the age, the worse the adherence in all areas studied (Table 2).

**Table 2:** Relationship of age and disease duration to adherence to ARMS treatment recommendations by patients with hypertension.

ARMS	Age		Duration of illness	
	*r*	*p*	*r*	*p*
ARMS overall score	0.505	<0.001	0.467	<0.001
Taking medication	0.504	<0.001	0.472	<0.001
Refilling of medicines and prescriptions	0.429	<0.001	0.368	<0.001

*p, coefficient of statistical significance; r, correlation coefficient.*

Based on the data collected, there were statistically significant differences in adherence to therapeutic recommendations according to the ARMS in individual domains by hypertensive patients depending on their place of residence. It was observed that the overall level of non-adherence to recommendations, as well as medication or medication refills and prescriptions, was significantly higher in patients from the largest cities than in the other study groups (Table 3).

**Table 3:** Adherence to therapeutic recommendations according to ARMS by hypertensive patients according to place of residence.

ARMS		Place of residence				p*
		Village A (*n* = 41)	City up to 10,000 flats. B (*n* = 39)	City of 10,000–100,000 inhabitants C (*n* = 82)	City over 100,000 inhabitants D (*n* = 43)	
ARMS overall score	M ± SD	21.80 ± 6.69	23.36 ± 5.78	24.52 ± 6.30	27.21 ± 6.13	<0.001C > A D > C, B,A
	Me	23	25	25	28	
	Q1-Q3	17.00–25.00	22.00–25.00	24.00–25.75	25.00–32.50	
Taking medication	M ± SD	13.90 ± 4.77	14.82 ± 4.20	15.78 ± 4.62	17.74 ± 4.62	<0.001C > A D > C, B, A
	Me	14	16	16	18	
	Q1-Q3	10.00–16.00	12.50–16.00	14.25–16.00	16.00–22.00	
Refilling of medicines and prescriptions	M ± SD	7.90 ± 2.14	8.54 ± 1.89	8.74 ± 1.80	9.47 ± 1.70	<0.001C > A D > C, B,A
	Me	8	9	9	10	
	Q1-Q3	6.00–9.00	8.00–9.00	9.00–9.00	9.00–11.00	

*Krukal-Wallis test + *post-hoc* analysis (Dunn’s test), SD, standard deviation; Q1, lower quartile; Q3, upper quartile; *p*, statistical significance coefficient,

On the basis of the collected data, statistically significant differences in adherence to therapeutic recommendations according to the ARMS were shown in the individual domains according to professional activity. The overall level of non-adherence (ARMS total score), the level of non-adherence to medication and to medication refills and prescriptions was significantly higher in inactive patients and pensioners than in active patients (Table 4). The study found no statistically significant relationships between the other sociodemographic variables (gender, marital status) and adherence to treatment among hypertensive patients (*p* > 0.05).

**Table 4:** Adherence to therapeutic recommendations according to ARMS by hypertensive patients according to work activity.

ARMS		Professional activity			p*
		Economically active - A (*n* = 47)	Inactive professionally - B (*n* = 13)	Pensioner - C (*n* = 145)	
ARMS overall score	M ± SD	19.98 ± 5.26	26.00 ± 6.54	25.58 ± 6.22	<0.001 B, C > A
	Me	22	25	25	
	Q1-Q3	14.5–24	23.0–26	25–30	
Taking medication	M ± SD	12.38 ± 3.62	16.92 ± 5.11	16.57 ± 4.55	<0.001 B, C > A
	Me	13	16	16	
	Q1-Q3	8–16	15–17	16–20	
Refilling of medicines and prescriptions	M ± SD	7.60 ± 1.93	9.08 ± 1.61	9.01 ± 1.82	<0.001 B, C > A
	Me	8	9	9	
	Q1-Q3	5.5–9	9–10	9–10	

*Kruskal-Wallis test + *post-hoc* analysis (Dunn’s test). SD, standard deviation; Q1, lower quartile; *p*, coefficient of statistical significance.

### 3.4 Adherence to therapeutic recommendations according to ARMS by patients diagnosed with hypertension according to medical variables

The study also assessed adherence to therapeutic recommendations according to ARMS by hypertensive patients according to medical variables (duration of disease, comorbidities, physical activity, dietary adherence).

There was a statistically significant association of adherence to treatment recommendations according to the ARMS by hypertensive patients in all domains and disease duration. This means that the longer the duration of the disease, the worse the adherence in all domains studied (Table 5). The study showed statistically significant differences in adherence to treatment recommendations according to the ARMS among patients with hypertension according to diabetes comorbidity. The overall level of non-adherence (ARMS total score), non-adherence to medication or medication refills and prescriptions was significantly higher in patients with diagnosed diabetes than in patients without diabetes (Table 5). In addition, statistically significant differences in adherence to treatment recommendations according to ARMS were observed among patients with hypertension according to the co-morbidity of circulatory insufficiency. The overall level of non-adherence, the level of non-adherence to medication and medication refills and prescriptions was significantly higher in patients without CHF than in patients with CHF.

**Table 5:** Adherence to therapeutic recommendations according to ARMS by hypertensive patients according to co-morbidity - diabetes mellitus, circulatory insufficiency or stroke.

ARMS		ARMS overall score			Taking medication			Refilling of medicines and prescriptions		
		M ± SD	Me	Q1-q3	M ± SD	Me	Q1-q3	M ± SD	Me	Q1-q3
Diagnosed diabetes	Yes (n = 88)	25.03 ± 6.21	25	24–28	16.19 ± 4.51	16	15–18	8.84 ± 1.84	9	9–10
	No (n = 117)	23.39 ± 6.69	25	20–25	14.90 ± 4.91	16	11.75–16	8.49 ± 2.02	9	7.75–10
*p*		0.008			0.009			0.169		
Circulatory insufficiency	Yes (n = 43)	22.02 ± 6.57	23	17–25	13.88 ± 4.81	14	09.05.2016	8.14 ± 1.97	9	7–9
	No (n = 162)	24.93 ± 6.31	25	23–26.75	16.10 ± 4.6	16	15–17	8.83 ± 1.89	9	9–10
*p*		0.004			0.002			0.038		
Stroke	Yes (n = 11)	26 ± 10.01	25	22–26	16.85 ± 7.57	16	14–17	9.18 ± 2.82	10	9–11
	No (n = 194)	24.23 ± 6.22	25	19.5–32.5	15.57 ± 4.53	16	10–22	8.66 ± 1.87	9	8–9.75
*p*		0.588			0.584			0.144		

SD, standard deviation; Q1, lower quartile; Q3, upper quartile; *p*, coefficient of statistical significance.

Based on the data collected, there were statistically significant differences in adherence to treatment recommendations according to ARMS among hypertensive patients according to physical activity. The overall level of non-adherence (ARMS total score) and medication adherence was significantly higher in physically inactive patients than in patients with ‘occasional’ activity, who in turn had a significantly higher level of non-adherence than in physically active patients. The level of non-adherence to medication refills and prescriptions was significantly higher in physically inactive patients than in physically active patients (Table 6).

**Table 6:** Adherence to therapeutic recommendations according to ARMS by hypertensive patients according to physical activity.

ARMS		Physical activity			*p*
		Every day. Several times per week - A (*n* = 18)	From time to time - B (*n* = 53)	At all - C (*n* = 134)	
ARMS overall score	M ± SD	20.17 ± 5.62	22.85 ± 6.81	25.46 ± 6.11	0.001C > B > A
	Me	22	25	25	
	Q1-Q3	14.25–24.75	17–26	24–27.75	
Taking medication	M ± SD	12.56 ± 4.02	14.62 ± 4.79	16.45 ± 4.56	0.001C > B, A
	Me	13.5	16	16	
	Q1-Q3	8–16	10–17	16–17	
Refilling of medicines and prescriptions	M ± SD	7.61 ± 1.94	8.23 ± 2.24	9.01 ± 1.69	0.008C > A
	Me	8.5	9	9	
	Q1-Q3	6–9	7–10	9–10	

SD, standard deviation; Q1, lower quartile; *p*, coefficient of statistical significance.

The study showed statistically significant differences in adherence to therapeutic recommendations according to the ARMS among hypertensive patients according to strict adherence to diet. It was observed that the overall level of non-adherence (ARMS total score) and the level of non-adherence to medication and prescription refills was significantly higher in patients not adhering to the diet or adhering “only sometimes” than in the group adhering strictly to the diet. Furthermore, the level of non-adherence to medication and prescription refills was also significantly higher in patients not adhering to the diet or adhering ‘only sometimes’ than in the strictly adhering group (Table 7).

**Table 7:** Adherence to therapeutic recommendations according to ARMS by hypertensive patients in relation to strictly observed diet.

ARMS		Strict adherence to the diet			*p*
		Yes - A (*n* = 29)	Sometimes - B (*n* = 130)	In general - C (*n* = 46)	
ARMS overall score	M ± SD	18.38 ± 7.14	25.39 ± 5.06	25.04 ± 7.57	<0.001 B,C > A
	Me	14	25	24.5	
	Q1-Q3	13–25	25–26	20.50–31.75	
Taking medication	M ± SD	11.59 ± 4.94	16.35 ± 3.83	16.17 ± 5.61	<0.001 B,C > A
	Me	8	16	16	
	Q1-Q3	8–16	16–17	12.25–21.75	
Refilling of medicines and prescriptions	M ± SD	6.79 ± 2.35	9.05 ± 1.43	8.87 ± 2.18	<0.001 B,C > A
	Me	6	9	9	
	Q1-Q3	5–9	9–9	8–10.75	

SD, standard deviation; Q1, lower quartile; Q3, upper quartile; *p*, statistical significance coefficient.

The study showed no statistically significant differences in adherence to treatment recommendations according to ARMS among hypertensive patients (*p* > 0.05) according to other medical variables (coexisting stroke or renal failure).

## 4 Discussion

A key prerequisite for effective chronic disease management is adherence to medical recommendations. Many organizations such as the World Health Organization-International Society of Hypertension (WHO-ISH) and the Polish Society of Hypertension (PTNT) (20) emphasize the role of patient-physician collaboration as a key aspect of treatment. Adherence to medical advice is therefore a key condition for effective therapy (22). Unfortunately, non-adherence to medical advice is a very common phenomenon that leads to serious health and economic consequences. Given the nature of the problem, many researchers have attempted to explain the cause of this phenomenon (23). Identifying the problem and understanding the reasons is crucial for decision-making and planning targeted interventions to reduce cardiovascular morbidity and mortality (24, 25) A review of the literature indicates that the level of patient adherence to medical recommendations is influenced by many factors, such as the type of disease and its severity, socioeconomic factors or the type of treatment, and there are also factors related to healthcare providers (27–29).

### 4.1 Adherence to medical recommendations among patients with hypertension

Our study demonstrated an average overall adherence score, indicating that non-adherence occurred “rarely.” Similarly, the subscales for “taking medicines” and “refilling medicines and prescriptions” also suggested that non-adherence to these specific recommendations was reported as “rarely.”

The few available studies suggest that adherence to antihypertensive treatment recommendations is often relatively low. A study by Naderi et al. found that adherence to cardiovascular disease prevention recommendations was only 57% (14). Similarly, a study in Italy found that about 60% of patients showed good or excellent adherence to antihypertensive treatment recommendations (32), whereas in Poland, only 26% of patients with cardiovascular disease adhered to medication recommendations (40). Many Asian studies mimic the same trend of low adherence to treatment recommendations; the percentage of patients showing good adherence was 66% in Vietnam (41), 65% among the Chinese population (42), 55% in Korea (43) and 53% in Malaysia (44). To sum up a review of the literature indicates that 30–50% of hypertensive patients do not take their medication as prescribed by their doctor (14, 15, 45–47).

The study by Andala et al. (48) found that only a small proportion of respondents exhibited high adherence to antihypertensive medication recommendations. In contrast, Bhusal et al. reported that the majority of patients demonstrated good adherence to antihypertensive treatment. A study conducted in Eastern Nepal, however, found that just over half of the patients adhered to antihypertensive therapy (49). Another study in Nepal by Khan et al. (50) showed notably lower adherence, with only a small proportion of patients following treatment recommendations. Similarly, research in Pakistan revealed that more than half of the participants were non-adherent. An African study also indicated that a significant portion of patients were poorly adherent to medication, which was strongly associated with uncontrolled blood pressure (51). Al-Daken’s study highlighted that a large group of participants reported generally good adherence (52). Furthermore, a meta-analysis by Mahmood et al., encompassing sixty-six studies from 22 Asian countries, estimated that the prevalence of non-adherence to antihypertensive medication recommendations in Asia was nearly 50 % (53).

### 4.2 Analysis of the effect of sociodemographic variables on adherence to treatment recommendations based on the ARMS questionnaire

Our own research has shown that some sociodemographic variables influence adherence to treatment recommendations by patients diagnosed with hypertension. It was observed that age positively correlates with ARMS total score, medication adherence and refill of medications and prescriptions. In addition, place of residence and occupational activity are significant predictors of adherence to treatment. It was noted that the overall level of non-adherence, as well as medication intake or medication and prescription refill, was significantly higher in patients from the largest cities than in the other study groups. Also, the overall level of non-adherence (ARMS total score), the level of non-adherence in terms of medication intake and in terms of medication and prescription refill was significantly higher in inactive patients, pensioners and pensioners compared to inactive patients. The study showed no significant statistical relationship between gender or marital status and adherence to treatment recommendations among hypertensive patients.

Different results were obtained in the studies by Pluta et al. (29) and Jankowska-Polańska et al. (36) where it was noted that gender influences the level of adherence to therapeutic recommendations. The authors showed that men had lower levels of adherence and compliance than women. On the other hand, Kulkarni et al. (30) observed that women were more likely than men to not adhere to medication recommendations.

A review of the literature indicates that non-adherence to medication decreases with age (54, 55) or does not affect adherence (56). In a study by Pluta et al. (29), it was found that age, duration of hypertension treatment and place of residence had no significant effect on adherence to treatment recommendations. In contrast, Rycombel et al. (38) observed that older people attached more importance to their health and fitness status, which positively influenced adherence and compliance levels. A study by Lor et al. (57) found that adherence scores decrease with age, indicating poor adherence to antihypertensive medication. These findings are in contrast to other studies, which showed that younger age: e.g. 18–44 years (58) or < 65 years (59) was associated with a higher risk of non-adherence to medication. On the other hand, Kardas et al. (37) noted that older age was associated with multimorbidity, which is often a cause of polypharmacy and thus influences non-adherence to medication.

Guo et al. (60) noted that a number of sociodemographic factors (e.g., age, education level, marital status) were significantly associated with adherence to medication among hypertensive patients. However, the impact of these factors on adherence remains unclear. Factors such as age, level of education and marital status may not directly influence the level of adherence to medical recommendations among hypertensive patients, but indirectly influence adherence to medical recommendations through the mediating role of health knowledge and/or social support.

### 4.3 Analysis of the effect of medical variables on adherence to treatment recommendations based on the ARMS questionnaire

Our own research has shown that certain medical variables influence adherence to treatment recommendations among patients diagnosed with hypertension. Analysis of the data showed that the duration of hypertension significantly correlates with the ARMS total score, medication adherence, and medication and prescription refills. In addition, it was observed that the overall level of non-adherence (ARMS total score), non-adherence to medication or prescription refills was significantly higher in patients with diagnosed diabetes than in patients without diabetes, and among patients without CHF than among patients with CHF. In contrast, the overall level of non-adherence to prescription and medication adherence was significantly higher in physically inactive patients than in patients with ‘occasional’ activity, who in turn had a significantly higher level of non-adherence than in physically active patients. The level of non-adherence to medication refills and prescriptions was significantly higher in physically inactive patients than in physically active patients. Also, adherence to dietary recommendations significantly affected adherence to therapeutic recommendations. It was observed that the overall level of non-adherence to recommendations and the level of non-adherence to medication and prescription refills was significantly higher in patients who did not adhere to diet or who adhered ‘only sometimes’ than in the strictly adherent group.

A review of the literature indicates that cognitive decline in older people may contribute to poorer adherence to medical recommendations (61, 62). Also, Rycombel et al. (38) noted that certain age-related illnesses, e.g., dementia, depression or Alzhaimer’s can reduce adherence and compliance. Similar results were obtained by Winnicki et al. (63) who noted that cognitive decline in older people affects adherence to therapeutic guidelines. In addition, they noted that duration of hypotensive treatment negatively affects adherence to treatment guidelines, most likely due to low patient persistence.

Also, a study by Jankowska-Polanska et al. (27), highlighted the significant influence of disease duration and thus treatment on adherence to hypertension therapy. In addition, Guo et al. (60) noted that among lifestyle factors, smoking, drinking alcohol, physical activity, vegetable consumption or regular physical activity, among others, significantly positively correlates with adherence to treatment among hypertensive patients. Yirga et al. (56) wrote that living far from follow-up health facility, absence of perceived symptoms, patient complaints Pill burdens, and poor Awareness about complication of hypertension were significantly associated with missing of their appointment follow-up for the most hypertensive patients.

The similar results presented Farah et al. (58) They wrote that several factors were significantly associated with higher adherence rates, including older age, higher educational level, regular blood pressure monitoring, and knowledge about their medications. Belayachi et al. (59) identified several risk factors contributing to non-adherence to medical recommendations. These included: moderate stress, lack of satisfactory family support, uncontrolled hypertension, depressive symptoms, insufficient patient-physician interaction, and inadequate medical management of cardiovascular conditions. Abza et al. (64) also discovered several variables that influence self-care adherence, including BMI, duration of antihypertensive treatment, number of antihypertensive medications, presence of comorbidities, and social support. Differences in study results may be due to cultural differences, financial disparities, and variations in health education across society. Moreover older patients in may already feel fatigued due to illness, and with age, adhering to medical recommendations becomes more challenging. This difficulty could be attributed to factors such as cognitive disorders, financial constraints, and limited access to medications.

## 5 Conclusion

The results from the ARMS questionnaire indicated that the majority of hypertensive patients in the study demonstrated good adherence to treatment recommendations, reflecting overall positive compliance. However, non-adherence remains a significant but often overlooked modifiable risk factor in the effective management of hypertension. The study highlighted that both patient-related factors and the role of medical staff are crucial in positively influencing adherence. Age, employment status, and place of residence were identified as key determinants, with older individuals, retirees, and those residing in larger urban areas exhibiting lower adherence levels. Consequently, targeted care and monitoring should be focused on hypertensive patients with a longer history of illness, those with comorbid diabetes, and individuals displaying poor health behaviors, such as insufficient physical activity and poor diet. These groups are more likely to experience non-compliance, and tailored interventions may be essential to improve adherence to prescribed treatment.

## 6 Limitation

Our study has some limitations. Firstly, many important variables related to medication adherence were included in the study; unfortunately, all known co-variables of medication adherence were not included (e.g., social support, cognitive status, therapy effect, dose of medication etc.). Secondly, the subjects were recruited from a single hospital and the sample size was small. Further research should be conducted in a multicentre setting with sufficient sample size to ensure that conclusions can be generalized. Thirdly, this study used a cross-sectional design, which precluded the possibility of inferring a causal relationship. Future longitudinal studies should be conducted to further verify causal relationships between variables.

## 7 Implication

Simple statement for healthcare providers:

- Healthcare providers should actively listen to patients’ stories and understand the contextual factors shaping their adherence behaviors. By doing so, they can develop more tailored and empathetic approaches to supporting adherence. Moreover, they should develop a process for routinely asking patients about medication adherence during consultations. Healthcare providers should consider nonadherence as a potential reason if a patient’s condition is not under control.
- Healthcare providers should create a shame- and blame-free environment to discuss medications with patients. They should encourage open dialog about doubts, concerns, and barriers to adherence. Moreover, they should identify why patients are not taking their medicine. Factors such as cognitive disorders, financial constraints, or lack of access to medications may contribute to nonadherence.

Policy Implications:

- Healthcare workers should adopt patient-centered approaches to treatment. Shared decision-making between patients and healthcare providers can lead to better adherence. Patients should be active partners in decisions about medication management, and their concerns and fears should be addressed.
- High levels of patient empowerment are associated with greater medication adherence. Healthcare providers should encourage patients to actively participate in their care decisions
- Healthcare providers should act in a sincere, empathetic manner, making patients feel comfortable and creating a safe space for discussion.
- Healthcare workers should remain present and mindful during care, addressing immediate concerns. They should incorporate preventative care to demonstrate concern for overall well-being.

## Data Availability

The raw data supporting the conclusions of this article will be made available by the authors, without undue reservation.
